# Aberrant functional connectivity and temporal variability of the dynamic pain connectome in patients with low back related leg pain

**DOI:** 10.1038/s41598-022-10238-4

**Published:** 2022-04-15

**Authors:** Yixiu Pei, Jidong Peng, Yong Zhang, Muhua Huang, Fuqing Zhou

**Affiliations:** 1 Department of Radiology, The First Affiliated Hospital, Nanchang University, 17 Yongwaizheng Street, Nanchang, Jiangxi 330006 People’s Republic of China; 2 Neuroradiology Laboratory, Jiangxi Province Medical Imaging Research Institute, Nanchang, 330006 People’s Republic of China; 3 Department of Pain Clinic, The First Affiliated Hospital, Nanchang University, Nanchang, Jiangxi 330006 People’s Republic of China; 4 Department of Medical Imaging, The Affiliated Ganzhou Hospital of Nanchang University, Ganzhou, Jiangxi 341000 People’s Republic of China

**Keywords:** Diseases, Signs and symptoms

## Abstract

Neuroimaging studies have suggested a link between the intensity of chronic low back pain intensity and structural and functional brain alterations. However, chronic pain results from the coordination and dynamics among several brain networks that comprise the dynamic pain connectome. Here, we use resting-state functional magnetic resonance imaging and measures of static (sFC) and dynamic functional connectivity (dFC) variability in the typical (0.01–0.1 Hz) and five specific (slow-6 to slow-2) frequency bands to test hypotheses regarding disruption in this variability in low back-related leg pain (LBLP) patients who experience chronic pain and numbness. Twenty-four LBLP patients and 23 healthy controls completed clinical assessments, and partial correlational analyses between altered sFC and dFC variability and clinical measures were conducted. We found a lower within-network sFC in the ascending nociceptive pathway (Asc) and a lower cross-network sFC between nodes of the salience network and the Asc in the typical frequency band. In the slow-5 frequency band, a lower within-network sFC was found in the Asc. Abnormal cross-network sFC was found between nodes of the salience network-Asc (slow-5 and slow-6) and the default mode network-Asc (slow-4 and slow-6). Furthermore, cross-network abnormalities in the typical and certain specific frequency bands were linked to clinical assessments. These findings indicate that frequency-related within- and cross-network communication among the nodes in the dynamic pain connectome is dysfunctional in LBLP patients and that selecting specific frequencies may be potentially useful for detecting LBLP-related brain activity.

## Introduction

Low back-related leg pain (LBLP) is a disease caused by intervertebral disc herniation and nerve root compression, which leads to low back pain or radiation pain of the lower extremities^1^ and seriously affects patients’ work and daily lives^2^. Some studies have found that approximately one-quarter to one-third of LBLP patients still have pain after surgery^3^, which may be related to the changes in central plasticity in patients with chronic LBLP^4–6^.

Pain signals are processed in different brain regions, especially in those corresponding to the “pain matrix”^7^. In essence, pain is a complex sensory and emotional experience, a process involving pain perception, emotion and cognition^8^. Nociceptive activity is a dynamic process in which some patients with chronic pain experience relief from feelings of pain when distracted from a painful stimulus. Hence, based on studies on the neural mechanisms of spontaneous attentional fluctuations and pain variability, Kucyi et al.^9^ introduced a new concept, the dynamic pain connectome, which is composed of the default mode network (DMN), salience network (SN) and descending antinociceptive pathway (Desc). Their study demonstrated that the relationship between the three systems is dynamic during the pain process and is crucial for the conduction of pain. Because it is essential for pain processing, a mismatch in the brain dynamics in crucial components of the pain connectome may form the basis of chronic pain conditions^9–13^.

The visual analog scale (VAS) is mainly used to evaluate the intensity of pain, but it is subjective and cannot represent the emotional, cognitive and dynamic changes of pain well^14^. However, functional magnetic resonance imaging (fMRI) can provide this multidimensional information better and thus may be the best way to observe the central nervous system of patients with pain in vivo. During the MRI scan, the blood-oxygenation-level-dependent (BOLD) signal oscillations change over the duration of the scan, and the BOLD signals in brain regions or networks with similar functions are correlated in time; this correlation is defined as the typical functional connectivity (FC). The typical static FC (sFC) characterizes the spatial pattern of the brain while ignoring the temporal variability in the BOLD signal. However, the relationship among the three systems that comprise the dynamic pain connectome is dynamic, and the endogenous characteristics of the brains of patients with pain may not be identified by using the sFC alone^9^. Dynamic functional connectivity (dFC) may reflect not only the intrinsic properties of the organization of the brain unrelated to the current cognitive state but also the spontaneous cognitive process of the subjects^9^. As a result, by examining the dFC, i.e., the variability in the BOLD signal, we can explore changes in the three networks in the dynamic pain connectome of patients with chronic LBLP.

BOLD signal fluctuations in different frequency bands may demonstrate different neurophysiological mechanisms^15^. Studies on fMRI generally consider the typical frequency band (0.01–0.1 Hz) to be of physiological significance^16,17^, but some studies of pain have found that the high frequency band (> 0.167 Hz) may also convey meaningful information^4,5,18–21^. Thus, the aim of this study was to determine whether the experience of persistent pain and paresthesia (numbness) influence the frequency-related cross- and within-network sFC and temporal variability in the dynamic pain connectome in LBLP patients. We hypothesize that the low- and high-frequency bands in the sFC and temporal variability in the dynamic pain connectome are impaired both cross- and within-network during the experience of pain and numbness in LBLP patients. Motivated by this hypothesis, we used seed-based correlational analysis to detect the within- and cross-network sFC in the dynamic pain connectome in the typical frequency band (0.01–0.1 Hz) and five specific frequency bands (to assess frequency specificity). We also examined frequency-related temporal variability in each region of interest (ROI) by sliding window correlational analysis and generated a time-varying coefficient of variation (CV) map to quantify the temporal variations in the FC. Then, we investigated whether these altered sFC and dFC variabilities were related to pain intensity and/or other indices.

## Materials and methods

### Subjects

The participants included 24 patients (mean age 51.35 ± 9.63 years, 14 males) diagnosed with LBLP and 23 healthy controls (HCs) with no significant difference in age or sex between the groups. All participants were recruited from the First Affiliated Hospital of Nanchang University and the community from October 2018 to July 2020, and all of them provided informed written consent to the procedures approved by the Medical Research Ethics Committee of the First Affiliated Hospital of Nanchang University in accordance with the Declaration of Helsinki.

To be included in the study, the patients had to meet the following criteria: (1) age 35–65 years; (2) a clear diagnosis of discogenic compression on lumbar computed tomography (CT) and/or magnetic resonance imaging (MRI) (> 1 ruptured annulus fibrosus with compressed soft tissue); (3) the sensation of radiating pain in the buttock(s) and lower limb(s) for more than 3 months and a visual analog scale (VAS) scores above 4; and (4) ineffective prior conservative treatment with medications, e.g., anti-inflammatory drugs (Motrin, Advil and Naproxen) and acetaminophen (e.g., Tylenol) without opioids, exercise and physical therapy. The exclusion criteria for the LBLP group were a history of spinal stenosis due to calcifications on the spinal protrusion, lateral recess stenosis, spinal stenosis, pyriformis syndrome, or sciatica, a history of head and spinal cord injury or a major systemic disease, and a history of significant cardiac events. For image quality control, participants with head motion exceeding a maximal translation < 3.0 mm and a maximal rotation < 3.0° were excluded.

### Clinical assessment

Clinical information obtained from the LBLP clinic included the VAS score, ranging from 0 to 10, for pain intensity, the Hamilton Depression Rating Scale (HAMD) and the Hamilton Anxiety Scale (HAMA) scores for evaluating the alterations of anxiety and depression in LBLP patients, the Mini-Mental State Examination (MMSE), reflecting the intellectual state and the degree of cognitive impairment of the participant, and the Japanese Orthopaedic Association (JOA) Back Pain Evaluation questionnaire (score range from − 6 to 29) for examining the impact of neuropathic or nociceptive pain on quality of life^22^.

### Imaging data acquisition

All participants in the study underwent a 3.0 Tesla MRI (Skyra, Siemens, Munich, Germany) scan. A high-resolution 3D-T1-weighted magnetization-prepared rapid gradient-echo (MP-RAGE) sequence (repetition time (TR)/echo time (TE) = 2530 ms/2.96 ms, field of view (FOV) = 256 mm × 256 mm, matrix = 256 × 256, thickness/gap = 1.0/0 mm and 176 sagittal slices) and a resting-state functional MRI (rs-fMRI) scan (TR/TE = 2000/30 ms, matrix = 64 × 64, FOV = 230 × 230 mm, 4-mm thickness, interslice gap of 0 mm, and 240 volumes over 8 min) were acquired. For the resting-state scan, participants were asked to “close your eyes and not fall asleep”.

Furthermore, we also collected additional conventional T2-weighted and fluid attenuated inversion recovery (FLAIR) sequences to determine anatomical brain abnormalities. For the diagnosis of LBLP, sagittal and axial conventional T1-weighted and T2-fat suppression sequences were performed (shown in the Supplementary materials).

### Preprocessing of fMRI data

All data processing was performed using the toolbox for Data Processing & Analysis of Brain Imaging^23^ (http://rfmri.org/dpabi) based on Statistical Parametric Mapping software (SPM12, http://www.fil.ion.ucl.ac.uk/spm/software/spm12/ ) run on the MATLAB 2014b platform (MathWorks, Natick, MA, USA).

The main preprocessing steps included removal of the first 10 volumes (20 s), slice timing and head motion correction, individual registration of high-resolution T1 images to echo planar imaging (EPI) images, segmentation from high-resolution T1 images templated by the Diffeomorphic Anatomical Registration Through Exponentiated Lie Algebra (DARTEL) toolkit followed by spatial normalization and transformation to Montreal Neurological Institute (MNI) space, resampling the imaging resolution to 3-mm isotropic voxels and spatial smoothing (6-mm full-width at half-maximum kernel), linear detrending and regressing out of nuisance variables, which included white matter, cerebrospinal fluid (CSF) and head motion parameters based on the Friston-24 model^24^. Notably, we did not perform global signal regression, as this could potentially distort the intrinsic functional connectivity at the group level and increase the negative correlations.

### Temporal filtering

Previous studies have indicated that the differential neurophysiological manifestations that underlie distinct frequencies may arise from neuronal input selection and plasticity^15,25^. To investigate the functional relationships in the dynamic pain connectome of patients with LBLP, we filtered the data to extract information from the typical frequency band (0.01–0.1 Hz), which was then divided into five specific frequency bands according to a previous study^26^: slow-6 (0–0.01 Hz), slow-5 (0.01–0.027 Hz), slow-4 (0.027–0.073 Hz), slow-3 (0.073–0.198 Hz) and slow-2 (0.198–0.25 Hz).

### Regions of interest

Seed-based correlational analysis was used to examine the functional connectivity in the dynamic pain connectome. The ROIs within key components of the dynamic pain connectome were chosen based on previous studies^27,28^, and are defined here using coordinates from MNI space: (1) DMN: posterior cingulate cortex (PCC) (− 2, − 46, 28) and medial prefrontal cortex (mPFC) (− 2, 50, 2); 2) SN: right temporoparietal junction (TPJ) (50, − 32, 28), right anterior insula (34, 18, 4), midcingulate cortex (MCC) (2, 12, 34), and right dorsolateral prefrontal cortex (dlPFC) (34, 46, 22); 3) Asc: left primary somatosensory cortex (S1) (− 34, − 30, 54), right S1 (34, − 28, 54), left secondary somatosensory cortex (S2) (− 60, − 30, 20), right S2 (60, − 22, 18), left posterior insula (− 34, − 20, 18), right posterior insula (34, − 20, 18); and 4) Desc: periaqueductal gray region (PAG) (0, − 32, − 10). For the connectivity analyses, we created a 6-mm sphere radius seed^29,30^ at the peak coordinates of the PCC, mPFC, right TPJ, right anterior insula, MCC, right dlPFC, and posterior insula, a 4-mm radius seed^6,31^ at the peak coordinates of the S1 and S2, and a 3-mm radius seed^11^ at the peak coordinates of the PAG.

### Static functional connectivity analysis

The BOLD signals within 230 time points at the 13 seeds were extracted for conducting Pearson correlation analysis, and the functional connectivity coefficients were calculated. Then, we performed a two-sample t test in SPSS software (version 21.0; IBM, Armonk, NY, USA) to assess the between-group differences in 78[13 × (13−1)/2] pairwise ROI z-FC values (*P* < 0.05) with false discovery rate (FDR) correction for multiple comparisons.

### Dynamic functional connectivity variability analysis

Dynamic functional connectivity variability analysis was conducted with a sliding time-window correlation method for each ROI, and the CV (SD/mean) map across time windows was computed. In brief, (1) a rectangular sliding window of length 20 TR (40 s) and step of 1 TR were selected to obtain windowed time series signals in accordance with previous studies^32,33^; (2) within 211 windows (for each ROI), 211 z-FC values were calculated, and the CV (SD/mean) map over time was computed to quantify the temporal variations in the FC; and (3) a two-sample t test was performed in SPSS to investigate the group differences in 78 [13 × (13–1)/2] pairwise ROI CV values (*P* < 0.05, uncorrected and FDR correction for multiple comparisons). Furthermore, different time window lengths (30 TRs and 50 TRs) and steps (2 TRs and 3 TRs) were set, and the temporal variability in the dynamic pain connectome across the two groups was investigated. The results are shown in Supplementary Table 1.

### Relationships with clinical measures

Partial correlation analyses were performed between individual sFC and CV values and clinical characteristics with age and sex as covariates.

## Results

### Demographics

The demographic information of the LBLP patients and HCs is summarized in Table 1.

**Table 1:** Demographic data and clinical characteristics of the LBLP group and HCs.

Characteristics	LBLP	Healthy controls	*P* values
	Mean (SD)	Mean (SD)	
Sex (M/F)	14/10	12/11	0.520^#^
Age (y)	51.35/9.63	50.63/8.71	0.788
VAS scores	5.21/0.98	n/a (n/a)	n/a
HAMD scores	24.91/5.43	n/a (n/a)	n/a
HAMA scores	5.75/3.55	n/a (n/a)	n/a
MMSE scores	6.71/4.97	n/a (n/a)	n/a
Total JOA scores	16.21/6.42	n/a (n/a)	n/a
Subjective symptoms	3.75/1.80	n/a (n/a)	n/a
Physical signs	3.88/1.36	n/a (n/a)	n/a
Daily activities	9.21/3.16	n/a (n/a)	n/a

*LBLP* low back-related leg pain, *F* female, *M* male, *n/a* not available, *SD* standard deviation, *JOA* Japanese orthopaedic association, *VAS* visual analog scale, *HAMD* Hamilton depression rating scale, *HAMA* Hamilton anxiety scale, Mini-Mental State Examination.

^#^Chi-squared tests.

### Static functional connectivity analysis: widespread within- and cross-network abnormalities in the typical and five specific frequency bands

**Figure 1:**
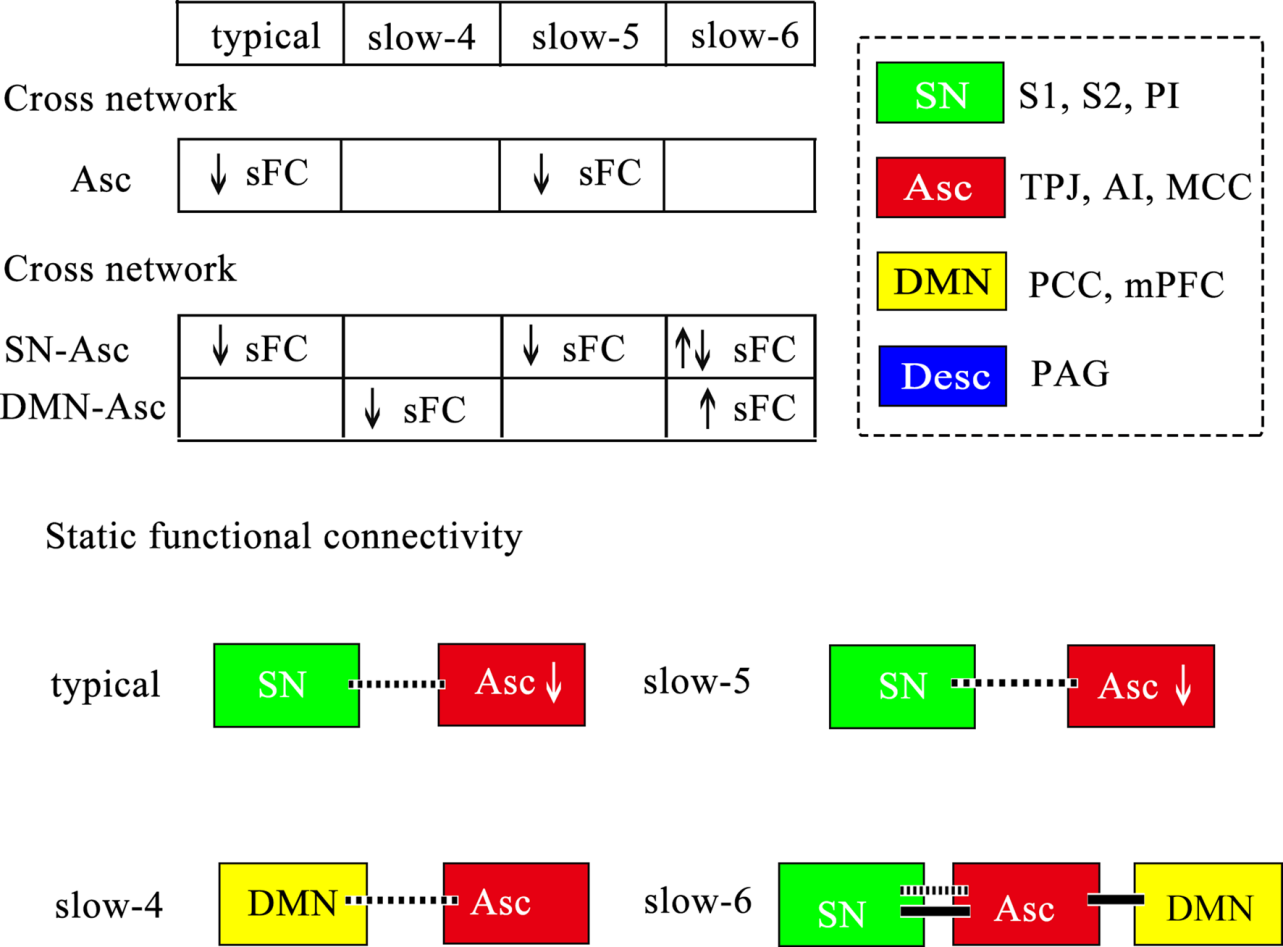
Summary figure showing within- and cross-network sFC and dFC variability abnormalities in chronic low back-related leg pain. Two-sample t test, *P* < 0.05, FDR correction. Note: The within- and cross-network abnormalities in the typical and five specific frequency bands are shown as a table in the figure. The right panel shows the legend for the dynamic pain connectome. Green indicates the salience network, blue indicates the descending antinociceptive pathway, and red indicates the ascending nociceptive pathway. Both static and dynamic functional connectivity variability are depicted in the figure. Solid lines indicate hyperconnectivity, and dotted lines indicate hypoconnectivity between networks. *sFC* static functional connectivity, *DMN* default mode network, *SN* salience network, *Asc* ascending nociceptive pathway.﻿

**Figure 2:**
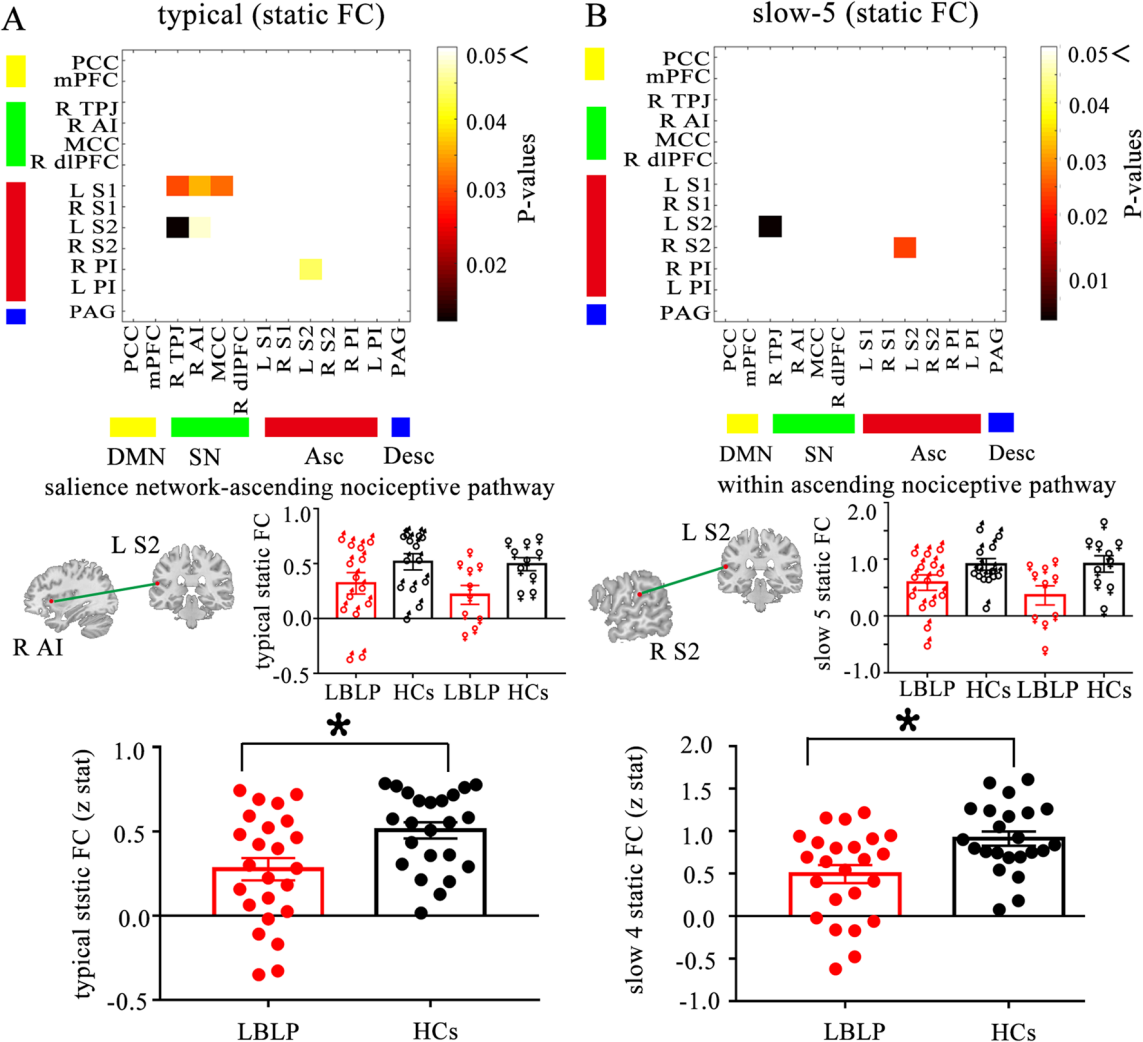
Static functional connectivity. Matrices indicate significant group differences in the typical (**A**) and slow-5 frequency bands (**B**). Two-sample t test, *P* < 0.05, FDR correction. Note: Examples of the sFC of the HCs and LBLP groups are shown below, where lines indicate the means ± standard error. *HCs* healthy controls, *LBLP* low back-related leg pain, *AI* anterior insula, *S2* secondary somatosensory cortex.

**Figure 3:**
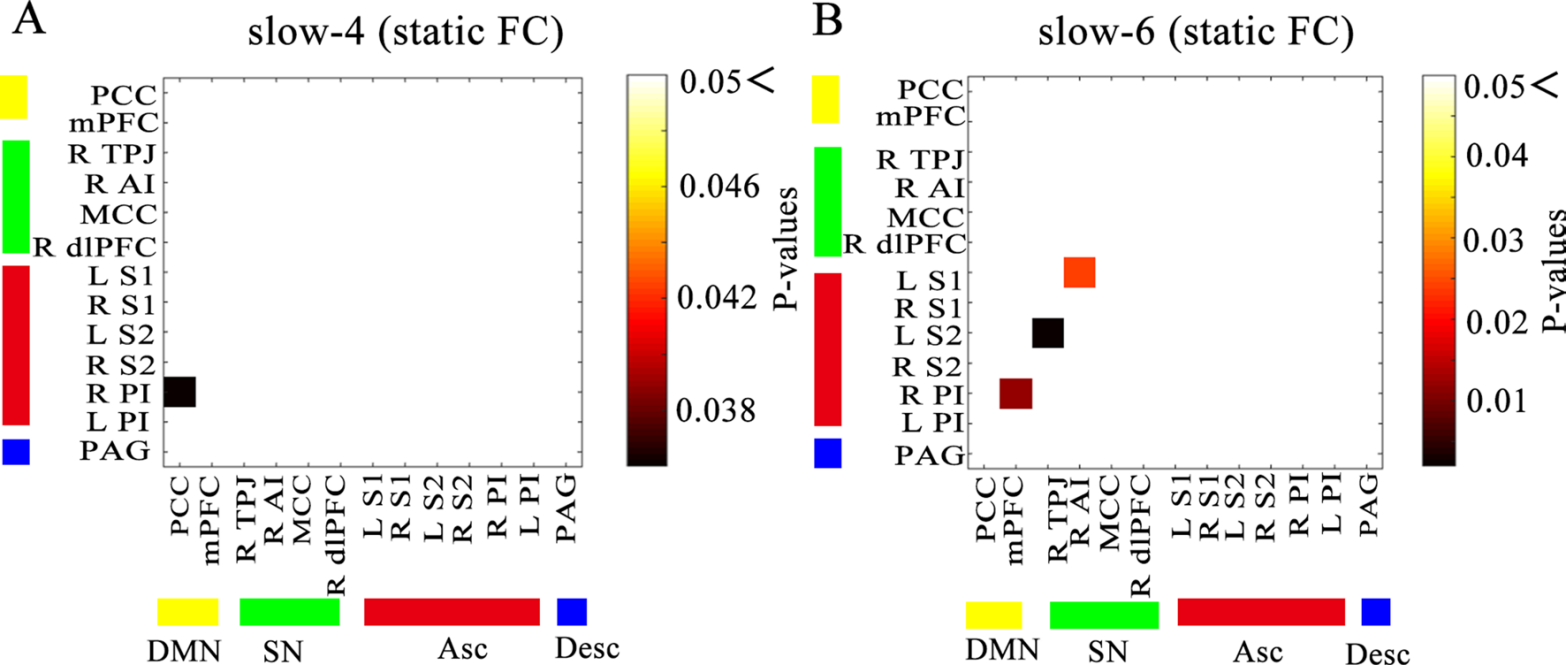
Static functional connectivity. Matrices indicate significant group differences in the slow-4 (**A**) and slow-6 (**B**) frequency bands. Two-sample t test, *P* < 0.05 with FDR correction.

Compared to the HCs group, the LBLP group exhibited abnormal sFC within and between the networks of the dynamic pain connectome in multiple frequency bands. Figure 1 shows a summary of these findings as assess with the two-sample t test (*P* < 0.05 with FDR correction). sFC values across all nodes and frequency bands for the LBLP patient and HCs groups are shown in Figs. 2 and 3.

In the sFC analysis of the typical frequency band, the chronic pain group exhibited lower within-network sFC between the core nodes of the Asc (right PI-left S2, *P* = 0.044) than the HCs group. Furthermore, the typical frequency band cross-network sFC was lower between nodes in the SN and Asc in the LBLP group than that in the HCs group (*P* = 0.012–0.048) (shown in Fig. 2A and see Supplementary Table 2).

In the sFC analysis of the five specific frequency bands, the within-network sFC was also lower in patients with LBLP than in HCs between the core nodes of the Asc (left S1-left S2, *P* = 0.024) in the slow-5 frequency band (shown in Fig. 2B and see Supplementary Table 3). There was also lower cross-network sFC between nodes of the SN and Asc (right TPJ-left S2, *P* = 0.012) in the slow-5 frequency band. However, the slow-6 frequency band demonstrated higher (right PI-left S1, *P* = 0.024) and lower (right TPJ-left S2, *P* = 0.024) cross-network sFC between nodes of the SN and Asc (shown in Fig. 3B and see Supplementary Table 3) and higher cross-network sFC between nodes of the DMN and Asc (mPFC-right AI, *P* = 0.012). Additionally, lower cross-network sFC was observed between nodes of the DMN and Asc (PCC-right PI, *P* = 0.036) in the slow-4 frequency band (shown in Fig. 3A and see Supplementary Table 3).

### Dynamic functional connectivity variability analysis: widespread within- and cross-network abnormalities in the typical and five specific frequency bands

We performed a two-sample t test for group differences in the dFC variability for each pairwise ROI. However, we did not find significantly different within- and cross-network dFC variability between the two groups after FDR correction.

Compared with the HCs, the LBLP group had higher dFC variability between the key nodes in the SN (right TPJ-MCC, *P* = 0.04, uncorrected) in the typical frequency band. Furthermore, the chronic pain group exhibited lower cross-network dFC variability between nodes of the SN and Asc (left S1-MCC, *P* = 0.014, uncorrected) (right anterior insula-left posterior insula, *P* = 0.047, uncorrected) and between nodes of the SN and Desc (right TPJ-PAG, *P* = 0.011, uncorrected) (shown in Supplementary Fig. 1A and Table 4).

However, higher dFC variability (slow-3 and slow-5) and lower dFC variability (slow-4) were found between areas of the Asc in the LBLP group (*P* < 0.05, uncorrected) (shown in Supplementary Fig. 1B and 2 and Table 4). Interestingly, we also found significant disease-related cross-network dFC variability differences in the five specific frequency bands mainly between the SN and Asc (*P* < 0.05, uncorrected).

### Subgroup analysis for sex differences among LBLP patients

**Figure 4:**
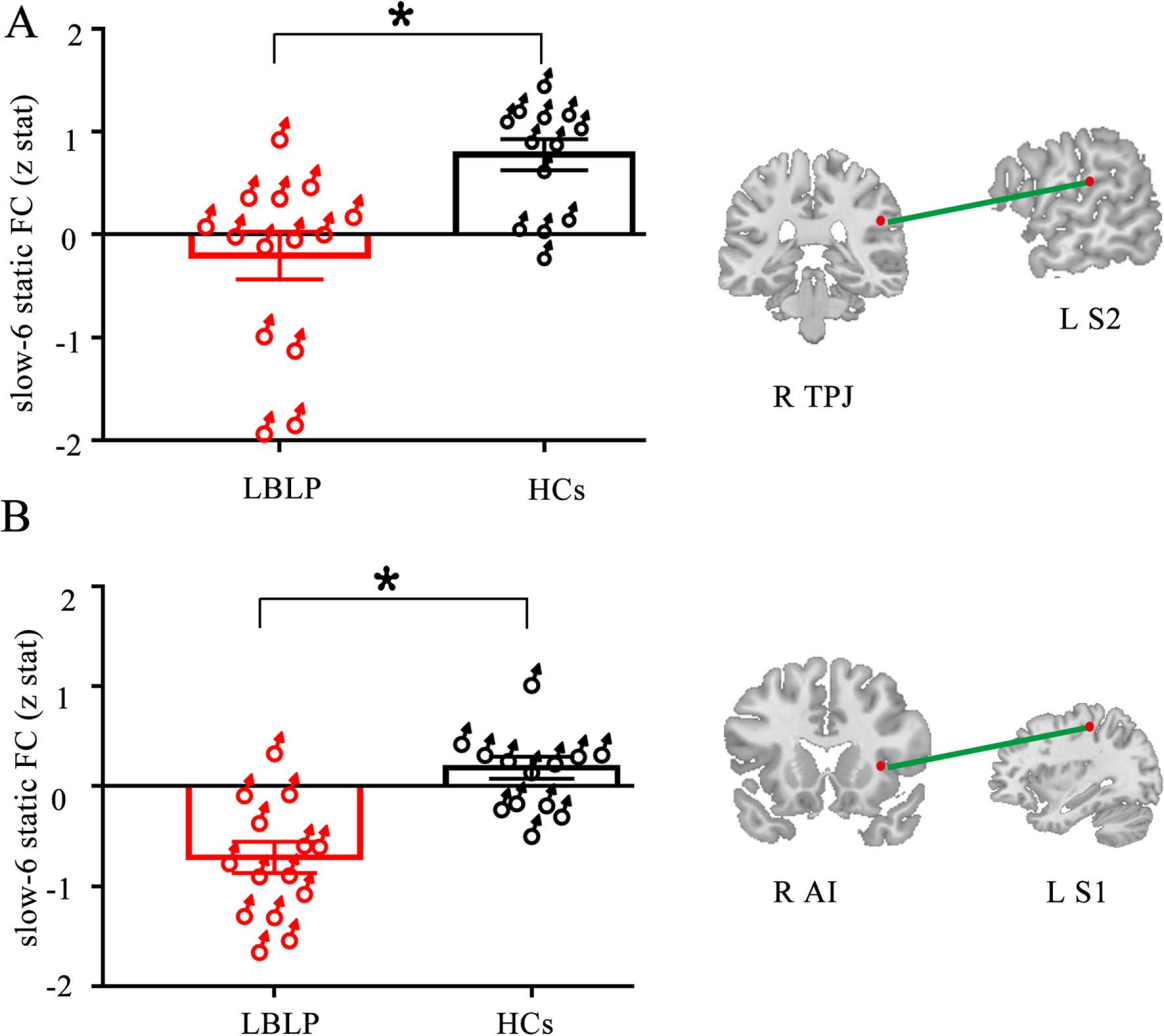
Male-specific analysis of differences between the LBLP and HCs groups. Two-sample t test, *P* < 0.05 with FDR correction.

**Figure 5:**
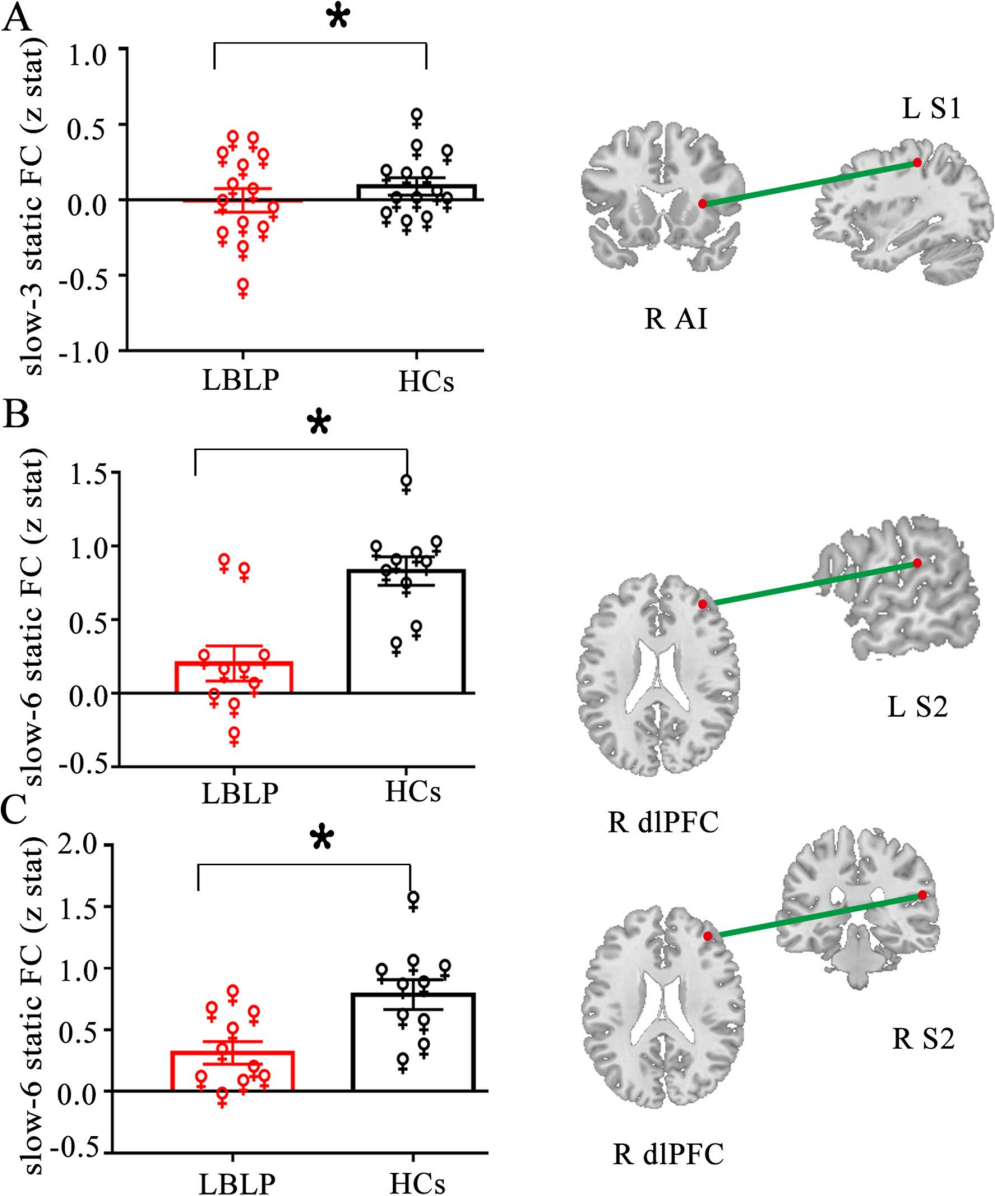
Female-specific analysis of differences between the LBLP and HCs groups. Two-sample t test, *P* < 0.05 with FDR correction.

To further explore the potential effects of sex on our results between LBLP patients and HCs, we compared the sFC and dFC variability in female and male patients relative to same-sex controls using a two-sample t test in SPSS (*P* < 0.05, FDR correction for multiple comparisons). In the male-specific studies, we found a lower cross-network sFC between the SN and Asc (right TPJ-left S2, *P* = 0.024) (right AI-left S1, *P* = 0.014) in the slow-6 frequency band (shown in Fig. 4). A lower cross-network sFC was also found between the SN and Asc (right AI-left S1, *P* = 0.012) (dlPFC-right S2, *P* = 0.014) in the slow-3 and slow-5 frequency bands in the female-specific studies (shown in Fig. 5). In both female- and male-specific studies, we did not find significantly different within- and cross-network dFC variability between the two groups after FDR correction.

### Relationships with clinical measures in LBLP patients

**Figure 6:**
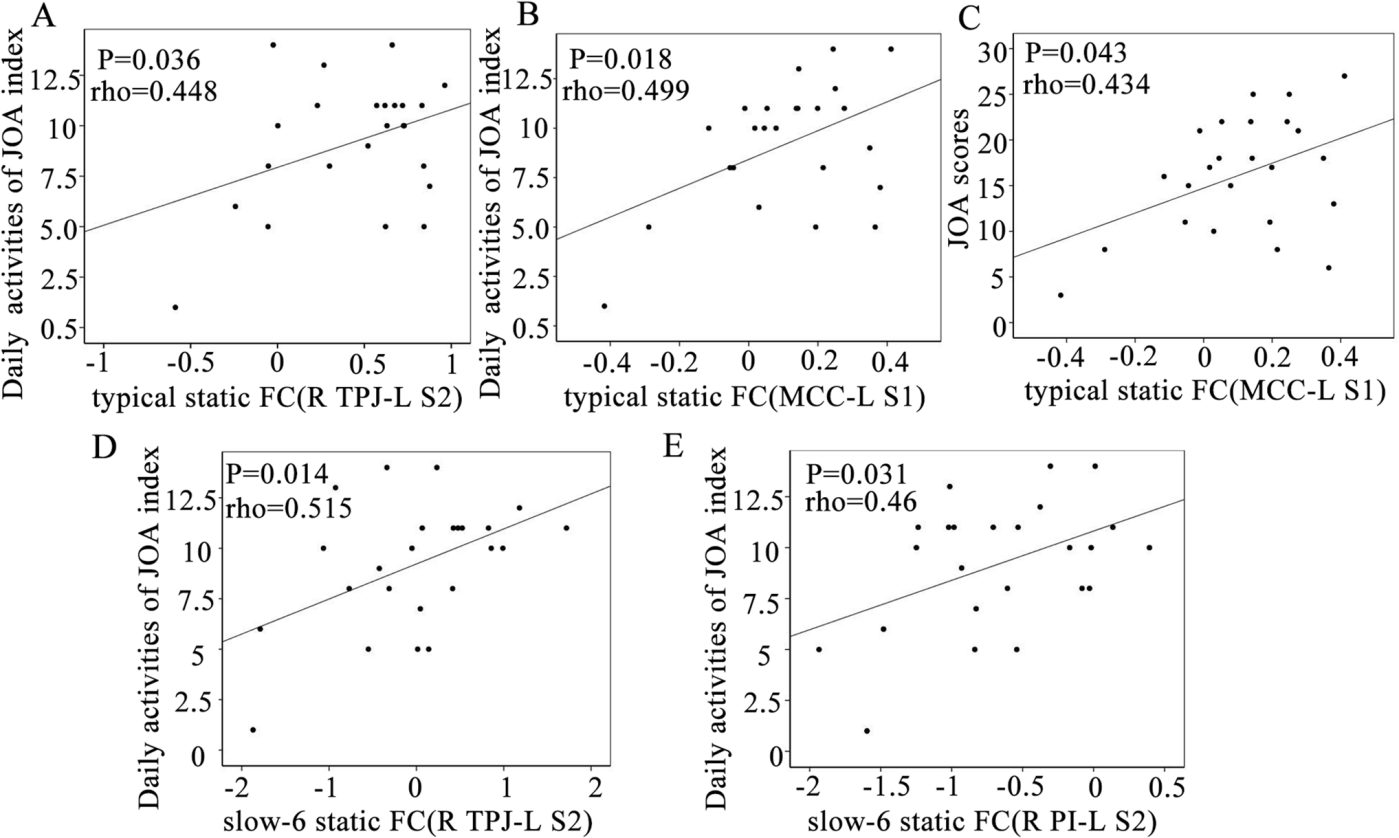
Partial correlational analysis between abnormal static FC and clinical assessment scores in LBLP patients. *JOA* Japanese orthopaedic association.

Within the LBLP group, the cross-network sFC values between the SN and Asc were significantly positively correlated with daily activities of the JOA index in the typical frequency band (right TPJ-left S2, rho = 0.448) (MCC-left S1, rho = 0.499) (shown in Fig. 6A–B). Additionally, the total JOA scores was positively correlated with the cross-network sFC between nodes of the SN-Asc (MCC-left S1, rho = 0.434) in the typical frequency band (shown in Fig. 6C). Finally, the cross-network sFC between nodes of the SN-Asc was positively correlated with daily activities of the JOA index in the slow-6 frequency band (right TPJ-left S2, rho = 0.515) (right PI-left S1, rho = 0.46) (shown in Fig. 6C–E). However, no correlations were found between altered sFC and any of the clinical indices (VAS, HAMD, HAMA and MMSE scores) in the typical and five specific frequency bands.

## Discussion

In this study, we demonstrated that patients with LBLP exhibit disrupted frequency-related within- and cross-network sFC and dFC variability in the dynamic pain connectome. Our main findings can be summarized as follows: (1) abnormalities were observed only in the sFC; (2) lower frequency-related within-network sFC was mostly located in the Asc; and (3) abnormal frequency-related cross-network sFC was mostly exhibited between nodes of the SN and Asc. Moreover, there was some pain-related abnormal cross-network sFC in the typical and specific frequency bands (slow-4, 5, 6) between the SN and Asc related to the JOA Back Pain Evaluation questionnaire scores. These main findings are mostly similar to those of a previous MEG study^27^ on chronic neuropathic pain in multiple sclerosis, indicating that frequency-related within- and cross-network communication is dysfunctional in patients with LBLP and demonstrating that selection of specific frequencies could be potentially useful for detecting LBLP-related brain activity.

### Within- and cross-network hypoconnectivity in LBLP patients in the typical and five specific frequency bands

We examined the within- and cross-network sFC to explore pain-related alterations in network organization in the typical frequency band and found lower within-network sFC located across nodes in the Asc. Interestingly, we also found cross-network hypoconnectivity in the typical band in the SN-Asc. The SN and Asc are the two networks that showed the most pronounced regional abnormalities in chronic pain, indicating that there may be a relationship between regional and interregional abnormalities in chronic pain^27,28^. In particular, the SN is associated with attention to an incoming stimulus, while the Asc is thought to transmit nociceptive inputs. In the current study, the lower within- and cross-network sFC in LBLP patients in the typical frequency band may have been associated with the persistent chronic pain state and attention to pain experienced by these patients.

In this study, the lower within-network sFC in the slow-5 frequency band was mostly located in the Asc, similar to that in the typical band. Notably, a lower sFC was found between nodes of the SN and Asc in the slow-5 band, but the sFC across the DMN-Asc nodes in the slow-4 frequency band was lower. The SN and DMN are anticorrelated networks in healthy subjects but become correlated in chronic pain^34,35^. Recent neuroimaging studies have found that physiological signals in different frequency bands are generated by different functional areas of the brain^25^. Physiological signals in the same brain network might compete or cooperate with each other in different frequency bands^36^. Our previous study showed that LBLP patients exhibit pain-related alterations in the cerebral amplitude of fluctuations and that regional homogeneity was frequency-dependent in several brain regions^5,18^. Thus, our study further proves that specific frequency ranges should be selected for the detection of pain-related intrinsic activity in LBLP patients. A common tactic for relieving pain is to divert attention, focusing on another matter attention elsewhere^37^. Furthermore, increased cognitive load can decrease pain perception^38^. Hence, we also found that the lower SN-Asc sFC in the typical and specific frequency bands was positively associated with the total JOA scores and the daily activities score of the JOA index in this study. This may indicate that cognitive load (paying more attention to pain) in LBLP patients could be increased, resulting in abnormal within- and cross-network sFC in the dynamic pain connectome.

### Alterations in dFC variability in LBLP patients in the typical and five specific frequency bands

As pain fluctuates over time, dynamic brain measurements within the dynamic pain connectome may capture the dynamics of pain perception^9^. In this study, although we did not find significantly different within- and cross-network dFC variability between the two groups after FDR correction, abnormal within- and cross-network dFC variability was identified between the two groups without multiple.

The sFC and dFC are complementary measures that can reflect different aspects of brain dynamics. In the dFC variability analysis, a higher dFC variability in interregional communication was observed mostly in the SN nodes in the typical frequency band. Regional BOLD signal variability is a sensitive indicator of individual differences in pain sensitivity and coping in healthy individuals^39^. Findings of increased within-network dFC variability but not sFC differences may be evidence of the ability of time-varying FC alterations to better reflect both spontaneous attentional fluctuations and intrinsic properties of brain organization related to chronic pain conditions^9^. However, patients exhibited similar cross-network dFC signal variability alterations to sFC in the typical frequency band. The lower dFC signal variability indicates that there is reduced flexibility in regional communication. The abnormal dynamic relationship between the SN and the Asc and Desc modulation pathways may reflect a change in the “balance between efficient information processing and metabolic expenditure”^40^. Furthermore, a higher dFC between the SN and the executive control network has been associated with better prioritization of cognitive tasks over pain stimuli^41^. Thus, patients with LBLP may focus more on pain, resulting in the inability of the brain to process pain information well.

Among the five specific frequency bands, we found higher within-network dFC variability between the nodes in the Asc in the slow-3 and slow-5 frequency bands. A previous study indicated that low-frequency oscillations play an important role in the context of identifying and evaluating the dFC^42^. The increased resting-state low-frequency oscillations within the Asc could be due to increased sensory communication as part of ongoing, fluctuating chronic pain intensity^41^. Notably, our cross-network dFC findings were distinct from the typical band results in that higher dFC variability was found across Asc-SN nodes in the four specific frequency bands (slow-2, slow-3, slow-5, and slow-6), but lower and higher dFC variability was found across the Asc-SN nodes in the slow-4 band. The pattern of intrinsic brain activity is sensitive to specific frequency bands^43^. Previous studies have demonstrated that the different oscillatory bands in the brain are generated by different mechanisms and possess different physiological functions^36^. Although the origin, relationship and specific physiological functions of the different frequency bands have not been fully clarified, it has been found that adjacent frequency bands within the same neuronal network are usually related to different brain states and compete with each other^25,36^. Of note, the frequencies subtended by the slow-5 and slow-4 bands are those typically utilized for typical frequency band analyses (0.01–0.1 Hz)^26^. We speculate that the slow-4 frequency band is the primary contributor to the lower dFC variability across SN-Asc nodes in the typical frequency band. Taken together, these findings suggest that dFC variability may be physiologically important in areas with relatively low- or high-frequency alterations and may reflect the regulation of disease status in patients with LBLP.

### Decreased sFC in female and male LBLP patients relative to same-sex controls

Neuroimaging studies have demonstrated that important sex differences are present in chronic pain, for instance, in the altered reactivity, morphology, and connectivity of major brain regions and networks involved in pain modulation^44,45^. However, mixed- and single-sex studies of chronic pain cannot provide a more comprehensive understanding of the commonalities and differences in brain alterations of female and male patients with chronic pain and may miss important findings^44^. Thus, it is necessary to directly compare men and women^46^. Previous studies have demonstrated that the gray matter of the primary motor and somatosensory cortex is altered more prominently in female patients with chronic pain^44,45,47,48^. In this study, a decreased cross-network sFC was found between the SN and Asc in male patients in the slow-6 frequency band, and a decreased cross-network sFC was found between the nodes of the SN and Asc in female patients in the slow-3 frequency band. Although our sample size is small, these findings nevertheless appear very interesting and important and further suggest that sex differences and their corresponding frequency attributes should be taken into account in chronic pain studies.

## Limitations

There are several limitations of this study. First, this is a relatively small sample size study. Thus, the interesting findings would need to be repeated to increase the statistical capacity with larger sample size studies and further research to discuss the effects of sex or gender. Second, we did not include acute-phase LBLP patients, and future expansion of the sample size to distinguish between acute, subacute and chronic LBLP groups could better reflect the impact of the disease on functional connectivity in the dynamic pain connectome. Finally, the potential physiological effects of medications on the BOLD fMRI signal were unclear in this study because the patients were not asked to stop taking them.

## Conclusion

In conclusion, the current study demonstrates the novel findings that patients with chronic LBLP exhibit frequency-related network-level abnormalities in the nodes in the dynamic pain connectome. Moreover, the clinical assessment scores in the typical and specific frequency bands correlated with alterations in the sFC and may provide a potentially useful approach to improve the detection of brain activity related to LBLP.

## Supplementary Information

Supplementary Information.
