# Supplementary material for: Aberrant functional connectivity and temporal variability of the dynamic pain connectome in patients with low back related leg pain
Source: Sci Rep. 2022 Apr 15;12:6324. doi: 10.1038/s41598-022-10238-4 (PMC9012841; doi:10.1038/s41598-022-10238-4)
Supplement: Supplementary file 1 — Supplementary Information. [file 41598_2022_10238_MOESM1_ESM.docx]

**Supplementary materials**

**Aberrant functional connectivity and temporal variability of the dynamic pain connectome in patients with low back related leg pain**

Yixiu Pei^1,2,4^, Jidong Peng^4^, Yong Zhang^3^, Muhua Huang^1,2^, Fuqing Zhou^1,2*^

^1^Department of Radiology, The First Affiliated Hospital, Nanchang University, Nanchang, 330006, PRC.

^2^Neuroradiology Laboratory, Jiangxi Province Medical Imaging Research Institute, Nanchang, 330006, PRC.

^3^Department of Pain Clinic, The First Affiliated Hospital, Nanchang University, Nanchang, Jiangxi Province, 330006, PRC.

^4^Department of Medical Imaging, The Affiliated Ganzhou Hospital of Nanchang University, Ganzhou, 341000, PRC.

*Correspondence: Dr. Fuqing Zhou

Department of Radiology, The First Affiliated Hospital, Nanchang University, 17 Yongwaizheng Street, Nanchang, Jiangxi 330006, China; Tel +86791 8869 5132; E-mail: [fq.chou@yahoo.com](mailto:fq.chou@yahoo.com)

**Supplementary 1:**

1. Conventional MRI sequences for determining anatomical brain abnormalities and for the diagnosis of the lumbar spine
2. Axial T2-weighted sequence (repetition time (TR)/echo time (TE) = 3000 ms/122 ms, field of view (FOV) = 240 mm × 240 mm, thickness/gap = 5.0/0 mm)
3. Axial T2-FLAIR sequence (TR/ TE = 3000 ms/122 ms, TI=2000 ms; FOV)= 240 mm × 240 mm, thickness/gap = 5.0/0 mm)
4. Conventional MRI sequences for the diagnosis of the lumbar spine

(1) Sagittal T1-weighted sequence (TR/ TE= 530 ms/7.2 ms, FOV = 250 mm × 250 mm, thickness/gap = 3.0/0 mm)

(2) Axial T1-weighted sequence (TR/TE) = 1000 ms/8 ms, FOV = 80 mm × 80 mm, thickness/gap = 7.0/0 mm)

(3) Sagittal T2-weighted sequence (TR/ TE) = 3314 ms/120 ms, FOV= 250 mm × 250 mm, thickness/gap = 3.0/0 mm)

(4) Axial T2-weighted sequence (TR/TE) = 4000 ms/120 ms, FOV = 80 mm × 80 mm, thickness/gap = 7.0/0 mm)

(5) T2 fat suppression sequence (TR/ TE) = 2200 ms/44 ms, TI=230 ms; FOV = 300 mm × 300 mm, thickness/gap = 3.0/0 mm)

**Supplementary Table 1**

**Altered dynamic functional connectivity at the typical frequency band between low back-related leg pain patients and healthy controls for different time window lengths (30 TR and 50 TR) and steps (2 TR and 3 TR) are shown below (two-sample t test, P < 0.05, uncorrected).**

| Brain regions  (dynamic FC) | LBLP (mean±SEM) | HCs (mean±SEM) | T values | P values | Effect size (Cohen's d) |
| --- | --- | --- | --- | --- | --- |
| *Altered dynamic FC at a window length of 20 TR and a step of 2 TR* | | | | | |
| mPFC-right dlPFC | 2.367±1.350 | -16.171±9.185 | -2.039 | 0.047 | 0.589 |
| MCC-right TPJ | 1.576±0.161 | 1.138±0.128 | -2.121 | 0.039 | 0.621 |
| Left S1-left PI | -3.524±1.906 | 4.813±3.538 | 2.099 | 0.041 | 0.609 |
| Left S2-left thalamus | 2.921±1.370 | -1.778±1.744 | -2.128 | 0.039 | 0.620 |
| *Altered dynamic FC at a window length of 20 TR and a step of 3 TR* | | | | | |
| MCC-right TPJ | 1.598±0.172 | 1.143±0.129 | -2.107 | 0.041 | 0.617 |
| Right dlPFC-left PI | -9.029±6.596 | 8.091±3.090 | 2.317 | 0.025 | 0.681 |
| Right S2-right S1 | -1.807±1.891 | 7.027±3.111 | 2.450 | 0.018 | 0.711 |
| *Altered dynamic FC at a window length of 30 TR and a step of 1 TR* | | | | | |
| Left S1-right dlPFC | -3.734±2.315 | 5.359±2.235 | 2.822 | 0.007 | 0.824 |
| Right PI-right S2 | 2.764±1.353 | -3.256±2.486 | -2.152 | 0.037 | 0.624 |
| Left thalamus-PAG | 6.032±2.252 | -2.026±1.682 | -2.848 | 0.007 | 0.834 |
| PAG-right dlPFC | -2.323±1.396 | 3.206±2.348 | 2.044 | 0.047 | 0.593 |
| PAG-left S1 | -1.819±1.433 | 2.388±0.757 | 2.563 | 0.014 | 0.753 |
| PAG-right thalamus | 0.502±0.694 | 5.224±1.844 | 2.436 | 0.019 | 0.705 |
| *Altered dynamic FC at a window length of 50 TR and a step of 1 TR* | | | | | |
| mPFC-MCC | 0.378±0.763 | 6.704±2.952 | 2.115 | 0.04 | 0.611 |
| Left S1-right thalamus | -1.247±0.974 | 1.579±0.576 | 2.471 | 0.017 | 0.725 |
| Right thalamus-mPFC | -1.098±5.488 | 2.145±0.763 | 2.373 | 0.022 | 0.695 |

*Note: FC, functional connectivity; LBLP, low back-related leg pain; HCs, healthy controls; mPFC, medial prefrontal cortex; dlPFC, dorsolateral prefrontal cortex; MCC, mid cingulate cortex; TPJ, temporoparietal junction; Sl, primary somatosensory cortex; PI, posterior insula; S2, secondary somatosensory cortex.*

**Supplementary Table 2**

**Significant alteration of the static functional connectivity at the typical frequency band between the LBLP patients and HCs (two-sample t test, P < 0.05, FDR correction).**

| Brain regions  (static FC) | LBLP (mean±SEM) | HCs (mean±SEM) | T values | P values | Effect size (Cohen's d) |
| --- | --- | --- | --- | --- | --- |
| *Altered static FC at the typical (0.01–0.1 Hz) frequency band (LBLP vs. HCs)* | | | | | |
| TPJ-left S1 | -0.032±0.076 | 0.244±0.055 | 2.923 | 0.03 | 0.856 |
| TPJ-left S2 | 0.444±0.084 | 0.803±0.058 | 3.472 | 0.012 | 1.017 |
| Right AI-left S1 | -0.141±0.054 | 0.102±0.054 | 3.186 | 0.036 | 0.930 |
| Right AI-left S2 | 0.276±0.067 | 0.506±0.048 | 2.781 | 0.048 | 0.815 |
| MCC-left S1 | 0.108±0.042 | 0.297±0.055 | 2.756 | 0.008 | 0.802 |
| Left S2-left PI | 0.057±0.057 | 0.293±0.069 | 2.645 | 0.044 | 0.770 |

*Note: FC, functional connectivity; LBLP, low back-related leg pain; HCs, healthy controls; M, male; F, female; TPJ, temporoparietal junction; MCC, mid cingulate cortex; S1, primary somatosensory cortex; S2, secondary somatosensory cortex; TPJ, temporoparietal junction; AI, anterior insula; PI, posterior insula.*

**Supplementary Table 3**

**Significant alteration of the static functional connectivity at the certain specific frequency bands between the LBLP patients and HCs (two-sample t test, P < 0.05, FDR correction).**

| Brain regions  (static FC) | LBLP (mean±SEM) | HCs(mean±SEM) | T-values | P-values | Effect size (Cohen's d) |
| --- | --- | --- | --- | --- | --- |
| *Altered static FC at the slow-4 (0.027-0.073 Hz) frequency band (LBLP vs. HCs)* | | | | | |
| PCC-left PI | 0.149±0.044 | 0.163±-0.009 | 3.198 | 0.036 | 0.094 |
| *Altered static FC at the slow-5 (0.01-0.027 Hz) frequency band (LBLP vs. HCs)* | | | | | |
| Right TPJ-left S2 | 0.511±0.110 | 1.017±0.074 | 3.775 | 0.012 | 1.106 |
| Left S2-right S2 | 0.494±0.106 | 0.912±0.084 | 3.077 | 0.024 | 0.900 |
| *Altered static FC at the slow-6 (0-0.01 Hz) frequency band (LBLP vs. HCs)* | | | | | |
| mPFC-right PI | 0.502±0.083 | 0.315±-0.174 | 3.672 | 0.012 | 0.628 |
| Right TPJ-left S2 | 0.617±0.179 | 0.728±-0.008 | 3.317 | 0.024 | 0.177 |
| Right AI-left S1 | 0.579±0.122 | 0.074±-0.664 | 4.305 | 0.024 | 1.174 |

*Note: FC, functional connectivity; LBLP, low back-related leg pain; HCs, healthy controls; M, male; F, female; mPFC, medial prefrontal cortex; S1, primary somatosensory cortex; PI, posterior insula; TPJ, temporoparietal junction; S2; secondary somatosensory cortex; AI, anterior insula; MCC, mid cingulate cortex; dlPFC, dorsolateral prefrontal cortex; PCC, posterior cingulate cortex; PAG, periaqueductal gray region.*

**Supplementary Table 4**

**Significant alteration of the dynamic functional connectivity at the typical and five specific frequency bands between the LBLP patients and HCs (two-sample t test, P < 0.05, uncorrected).**

| Brain regions  (dynamic FC) | LBLP (mean±SEM) | HCs (mean±SEM) | T values | P values | Effect size (Cohen's d) |
| --- | --- | --- | --- | --- | --- |
| *Altered dynamic FC at the typical (0.01–0.1 Hz) frequency band (LBLP vs. HCs)* | | | | | |
| MCC_right TPJ | 1.562±0.155 | 1.135±0.128 | -2.113 | 0.040 | 0.618 |
| Right TPJ-PAG | -2.229±1.581 | 3.379±1.406 | 2.643 | 0.011 | 0.772 |
| Right AI-right PI | -1.524±2.218 | 11.790±6.245 | 2.043 | 0.047 | 0.591 |
| Left S1-right TPJ | -1.496±1.377 | 2.998±1.061 | 2.570 | 0.014 | 0.752 |
| *Altered dynamic FC at the slow-6 (0–0.01 Hz) frequency band (LBLP vs. HCs)* | | | | | |
| mPFC-left S1 | -7.687±4.152 | 2.814±1.498 | 2.339 | 0.024 | 0.688 |
| Right TPJ-right thalamus | 2.498±1.083 | -3.961±2.975 | -2.074 | 0.044 | 0.600 |
| Right TPJ-PAG | -1.488±1.641 | 3.519±1.460 | 2.273 | 0.028 | 0.664 |
| *Altered dynamic FC at the slow-5 (0.01–0.027 Hz) frequency band (LBLP vs. HCs)* | | | | | |
| Right S1-right TPJ | 3.627±1.250 | -0.566±1.507 | -2.149 | 0.037 | 0.626 |
| Right S2-left PI | 2.792±1.200 | -0.424±1.008 | -2.043 | 0.047 | 0.598 |
| Left PI-right PI | 1.233±1.048 | -3.679±1.935 | -2.258 | 0.029 | 0.655 |
| PAG-left PI | -3.195±1.615 | 5.349±3.266 | 2.375 | 0.022 | 0.689 |

*Note: FC, functional connectivity; LBLP, low back-related leg pain; HCs, healthy controls; M, male; F, female; MCC, mid cingulate cortex; TPJ, temporoparietal junction; PAG, periaqueductal gray region; AI, anterior insula; PI, posterior insula; S1, primary somatosensory cortex; mPFC, medial prefrontal cortex; dlPFC, dorsolateral prefrontal cortex; S2; secondary somatosensory cortex; PCC, posterior cingulate cortex.*

**Refer to Supplementary Table 4 (continued)**

**Significant alteration of the dynamic functional connectivity at the typical and five specific frequency bands between the LBLP patients and HCs (two-sample t test, P < 0.05, uncorrected).**

| Brain regions  (dynamic FC) | LBLP (mean±SEM) | HCs (mean±SEM) | T values | P values | Effect size (Cohen's d) |
| --- | --- | --- | --- | --- | --- |
| *Altered dynamic FC at the slow-4 (0.027-0.073 Hz) frequency band (LBLP vs. HCs)* | | | | | |
| Right TPJ-right PI | 11.693±6.409 | -4.020±4.043 | -2.053 | 0.046 | 0.602 |
| Right TPJ-PAG | -1.996±1.558 | 2.701±1.176 | 2.390 | 0.021 | 0.700 |
| mPFC-right dlPFC | 7.909±4.331 | -24.467±12.731 | -2.449 | 0.018 | 0.708 |
| Left S1-left thalamus | -0.780±2.074 | 7.150±3.234 | 2.082 | 0.043 | 0.605 |
| Right S1-right PI | -7.986±5.339 | 9.528±5.133 | 2.362 | 0.023 | 0.690 |
| Right S2-right thalamus | 8.446±4.107 | -1.101±1.501 | -2.147 | 0.037 | 0.632 |
| Right AI-left thalamus | 2.797±0.772 | -3.487±2.764 | -2.230 | 0.031 | 0.645 |
| Right PI-left thalamus | -3.514±3.612 | 5.548±2.452 | 2.058 | 0.045 | 0.603 |
| Left thalamus-PAG | -2.352±1.799 | 13.831±7.491 | 2.142 | 0.038 | 0.619 |
| Right AI-right thalamus | -4.638±2.978 | 3.979±2.458 | 2.222 | 0.031 | 0.650 |
| Left thalamus-right thalamus | 0.924±0.137 | 1.814±0.406 | 2.113 | 0.040 | 0.611 |
| *Altered dynamic FC at the slow-3 (0.073-0.167 Hz) frequency band (LBLP vs. HCs)* | | | | | |
| PCC-MCC | 6.079±2.457 | -0.697±1.699 | -2.250 | 0.029 | 0.659 |
| Right AI-left S1 | 11.989±7.301 | -5.476±3.500 | -2.127 | 0.039 | 0.625 |
| Left S1-PAG | -1.231±2.057 | 7.422±3.336 | 2.229 | 0.031 | 0.647 |
| Right S2-right PI | 4.579±1.991 | -0.660±1.483 | -2.096 | 0.042 | 0.614 |
| *Altered dynamic FC at the slow-2 (0.167-0.25 Hz) frequency band (LBLP vs. HCs)* | | | | | |
| Right TPJ-left thalamus | 4.949±1.795 | -1.238±1.398 | -2.704 | 0.010 | 0.791 |


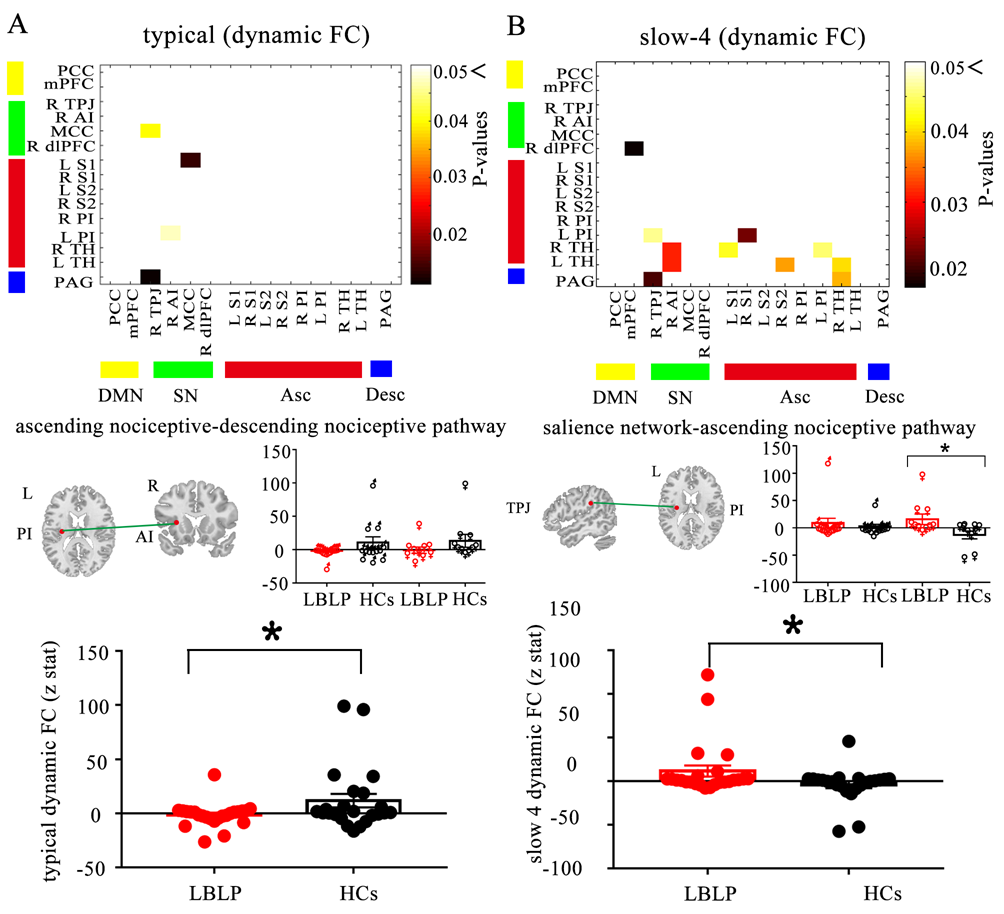


Supplementary Figure 1. Dynamic functional connectivity. Matrices indicate significant group differences in the typical (A) and slow-4 frequency bands (B). Two-sample t test , P < 0.05, uncorrected.

*Examples of static functional connectivity for the HCs and LBLP groups are shown below, and lines indicate the means ±standard error. AI, anterior insula; TPJ, temporoparietal junction.*


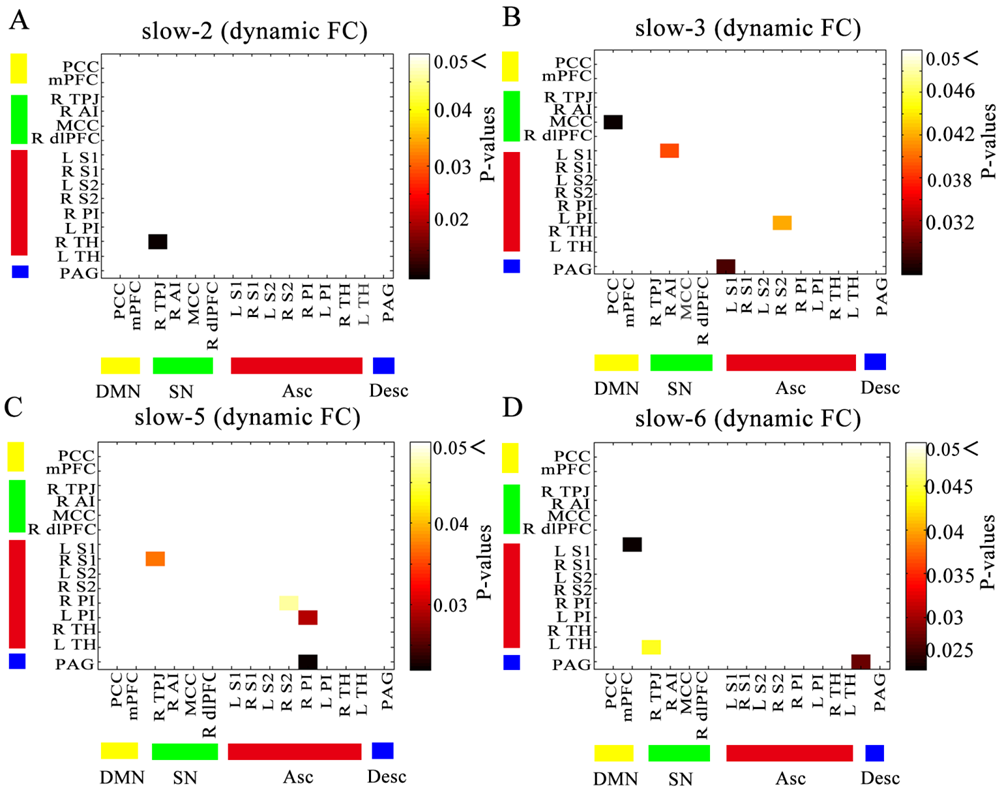


Supplementary Figure 2. Dynamic functional connectivity. Matrices indicate significant group differences in the slow-2 (A), slow-3 (B), slow-5 (C) and slow-6 frequency bands (D). Two-sample t test , P < 0.05, uncorrected.
